# Diethylene glycol: Unnoticed threat in the landscape of fixed-dose combination medications

**DOI:** 10.4102/jphia.v16i1.1271

**Published:** 2025-04-24

**Authors:** Alemayehu L. Duga, Mosoka P. Fallah, Albert Figueras

**Affiliations:** 1 Africa Centres for Disease Control and Prevention, Addis Ababa, Ethiopia; 2 Department of Pharmacoepidemiology and Pharmacovigilance, Doctoral School Societies, Politics, Public Health, University of Bordeaux, Bordeaux, France

**Keywords:** diethylene glycol, ethylene glycol, Irrational medicine use, irrational fixed-dose combination, pharmacovigilance

## Abstract

Diethylene glycol (DEG) and ethylene glycol (EG) are organic compounds often found in various consumer products, including antifreeze and industrial solvents used in pharmaceutical preparations, as well as serving as raw materials for polymer manufacturing. Since September 2022, seven consecutive episodes of DEG and EG contamination have been reported across at least nine countries. A notable commonality among the affected products is that at least 14 of them are fixed-dose combinations (FDCs). However, the evidence supporting the efficacy of most of these combinations is insufficient, which renders their prescription, dispensing, and use irrational. Moreover, these products are not without risk, as they can cause adverse reactions. Several factors contribute to the prevalence of these irrational FDCs, including low production costs, consumer popularity, and a tendency to authorise locally manufactured products. As a result, many countries’ pharmaceutical markets keep marketing authorisation for irrational FDCs. The persistent reports of DEG or EG contamination – especially those involving irrational fixed-dose combinations – present a crucial opportunity to enhance quality control measures. In addition, it is imperative to reevaluate the marketing authorisations of these products that lack evidence of safety and efficacy, adapting the national medicines lists and clinical guidelines to WHO recommendations. Strengthening regulatory frameworks and implementing stringent manufacturing and quality assurance standards are essential to prevent contamination incidents and ensure the safety of pharmaceutical products.

## Introduction

Diethylene glycol (DEG) and ethylene glycol (EG) are organic compounds found in some consumer products like antifreeze and industrial solvents and as a raw material for manufacturing polymers. In 1937, the Elixir Sulfanilamide disaster was one of the most consequential mass poisonings of the 20th century. It occurred shortly after introducing sulfanilamide, the first sulfa antimicrobial, when DEG was used as the diluent to formulate a liquid preparation of Elixir Sulfanilamide.^1^ It produces acute kidney injury (AKI), which may sometimes be fatal, and children are the most affected population, as DEG is used for the formulation of syrups or other oral solutions.^2^ In response to this significant public health crisis, the United States Congress enacted the *Federal Food, Drug, and Cosmetic Act of 1938*.^3^ This legislation established the requirement for demonstrating the safety of new drugs prior to their market release.^4^

Diethylene glycol has been linked to numerous mass poisoning events since 1937, often because of its inadvertent use in pharmaceuticals. These incidents primarily occur in developing nations with limited medical resources. For instance, between 1990 and 1998, DEG poisoning in countries such as Argentina, Bangladesh, India and Nigeria led to 100 of deaths.^5^ In the United States, a review of ethylene glycol exposures from 2006 to 2013 recorded 45 097 cases and 154 fatalities.^6^ Ethylene glycol is metabolised into toxic compounds, causing metabolic acidosis, renal damage and neurotoxicity.^7^

Diethylene glycol-associated tragedies have been reported multiple times. Since September 2022, seven consecutive episodes of this poisoning involving different products have been reported in nine countries (Table 1). Analyses of these cases indicate that lapses in the production process of these medicinal syrups result in mishaps.^1^ These include bypassing quality checks of raw products, mislabelling product shelf life, and missing records and logbooks, among others.^8^ World Health Organization (WHO), including other agencies and national medicines authorities, have carefully analysed each of these cases and their causes to implement actions to prevent new episodes. Certainly, improving the quality control systems, auditing local manufacturers and imported medicines, avoiding the informal medicines market and fighting counterfeit and substandard products are pillars to reduce the chance of new episodes.

**TABLE 1:** Most relevant episodes of intoxications after taking products contaminated with diethylene glycol, reported product composition, fixed-dose combination condition, and listing to the 2023 World Health Organization essential medicines list.

Date	Country	Reported preparation	Ingredients including excipients	FDC	WHO EML
**Recent episodes 2022–2024**					
December 2023	Pakistan	Mucorid syrup	Ambroxol + Salbutamol + Guaifenesin + Menthol	√	n/a
		Ulcofin suspension	Famotidine	n/a	√
		Alergo syrup	Cetirizine Di-Hcl	n/a	√
		Emidone suspension	Domperidone	n/a	√
		Zincell	Zinc	n/a	√
August 2023	Iraq	COLD OUT syrup	Paracetamol + Chlorpheniramine Maleate	√	n/a
July 2023	Cameroon	Naturcold syrup	Paracetamol + phenylephrine hydrochloride + chlorpheniramine maleate	√	n/a
April 2023	Marshall Islands/Micronesia	Guaifenesin syrup TG	Guaifenesin	n/a	n/a
January 2023	Uzbekistan/Cambodia	Ambronol syrup	Ambroxol	n/a	n/a
		DOK-1 Max syrup	Paracetamol BP + Guaifenesin BP + Phenylephrine Hydrochloride BP	√	n/a
November 2022	Indonesia	Termorex syrup (batch AUG22A06 only)	Paracetamol	n/a	√
		Flurin DMP syrup	Paracetamol + Pseudoephedrine HCl + Dextromethorphan HBr + Chlorpheniramine Maleate	√	n/a
		Unibebi Cough syrup	Paracetamol + Guaifenesin + Chlorphenamine Maleate	√	n/a
		Unibebi Demam Paracetamol drops	Paracetamol	n/a	√
		Unibebi Demam Paracetamol syrup	Paracetamol	n/a	√
		Paracetamol drops PT Afi Farma	Paracetamol	n/a	√
		Paracetamol syrup (mint) PT Afi Farma	Paracetamol	n/a	√
		Vipcol syrup	Paracetamol + Guaifenesin + Chlorphenamine Maleate	√	n/a
October 2022	The Gambia	Promethazine oral solution	Promethazine	n/a	n/a
		Kofexmalin Baby Cough syrup	Pheniramine Maleate + Ammonium chloride + Menthol	√	n/a
		Makoff Baby Cough syrup	Chlorphenamine Maleate + Phenylephrine HBr + Dextromethorphan	√	n/a
		Magrip N Cold syrup	Paracetamol + Phenylephrine HCl + Chlorphenamine Maleate	√	n/a
**Episodes 1937–2020**					
2020	India	Coldbest cough syrup	Paracetamol + Phenylephrine HCl + Chlorpheniramine maleate	√	n/a
2008	Nigeria	My Pikin Baby Teething Mixture	Not informed	√	n/a
2006	Panama	CSS Jarabe antitusigeno	Cough mixture manufactured by the national laboratory including an ‘expectorant’ plus and ‘antihistamine’	√	n/a
2006	India	Paracetamol syrup	Paracetamol	n/a	√
1998	India	Enchest expectorant	Bromhexine HCl + Pseudoephedrine HCl	√	n/a
		Decoryl suspension	Phenylephrine HCl + Paracetamol + Chlorpheniramine maleate	√	n/a
1995–1996	Haiti	Paracetamol syrup	Paracetamol	n/a	√
1990–1992	Bangladesh	Paracetamol suspension	Paracetamol	n/a	√
1990	Nigeria	Paracetamol syrup	Paracetamol	n/a	√
1937	US	Sulfanilamide elixir	Sulfanilamide	n/a	n/a

*Source:* Adapted from World Health Organization. eEML – Electronic essential medicines list [homepage on the Internet]. 2020 [cited 2025 Feb 14]. Available from: https://list.essentialmeds.org/

Note: √ in the FDC column reports the preparation containing two or more active ingredients in one formulation. √ in the WHO EML column reports the preparation is listed in the World Health Organization’s essential medicines list.

FDC, fixed-dose combination; WHO EML, World Health Organization essential medicines list; US, United States; n/a, not applicable.

## Strengthening detection and prevention systems for diethylene glycol -contaminated medicines

Implementing all the necessary controls in the medicines value chain is complex it takes time, and requires certain investment in audit means and training personnel, among other needs. All these processes can be muddled because DEG is a colourless, practically odourless, hygroscopic liquid with a sweetish taste, which increases the chance of human error.^2^ Introducing a mandatory striking colourant or odour to DEG would not only help differentiate it from consumable liquids but also serve as an immediate visual or olfactory alert during manufacturing and handling processes. This simple yet effective measure could significantly mitigate the risk of accidental contamination and prevent such incidents from escalating into public health crises.^9^

In theory, periodic internal audits by manufacturers and strong inspection programmes by health authorities should be enough to detect almost all these cases. The purity of glycerol can be tested using relatively cheap technology such as refractometry.^10^ Notwithstanding this, new cases of substandard medicines reappear, and healthcare professionals and consumers feel unsure about the quality of medicines.

The repeated DEG episodes result from a double failure. Firstly, the system has been unsuccessful in preventing these products from reaching the market. Secondly, there is a lack of capacity for the early detection of these products if they end up in pharmacies or street markets. Considering this situation, beyond the mandatory tests conducted by manufacturers and the strict inspections designed to identify any human error or negligence, it is also essential to focus on accelerating the identification and investigation of any suspected cases. This approach will help to reduce the number of patients affected.

Poor-quality medicines particularly affect low-income countries, where information and drug regulation enforcement are scant; additionally, inadequate infrastructure, non-regulated drug outlets, and informal market operations make medicines quality surveys difficult.^11^ In order to mitigate the harms produced by substandard products in general and those contaminated with DEG in particular, the best response consists of strengthening quick detection systems to avoid affecting many patients before the alarm is raised.

### Diethylene glycol poisoning risks in fixed-dose combinations excluded from World Health Organizationessential medicines

A review of the cases of reported DEG poisoning highlighted in Table 1 shows one factor common to the products involved in the accidents: 14 of the involved products were fixed-dose combinations marketed for the symptomatic treatment of the common cold or flu. The evidence supporting most of these combinations is lacking, so their prescription, dispensing, and use are irrational.^12^ Furthermore, these products are not devoid of adverse reactions. This finding is a concern as several local manufacturers, despite having a limited production infrastructure and Research and Development (R&D) departments, have started the development of FDCs as a popular lifecycle-management strategy to maximise the value of their products. The development of these FDCs is driven by factors such as the patent expiry of major blockbuster drugs, wide acceptance from consumers as a ‘one-stop’ solution to treat cold symptoms, stiffer competition from generic drug makers, and a drought in the pharmaceutical R&D pipeline.^13^ The opportunity, affordable production costs, popularity, and perhaps a tendency to authorise locally manufactured products make it possible for many countries’ pharmaceutical markets to keep marketing authorisation for many of these FDCs.^14^

Although the development of FDCs is important from a public health perspective, they must be shown to be safe and effective for the claimed indications. In addition, there should be clear clinical benefits in the form of increased efficacy and a reduced incidence of adverse effects.^15^ It should be remembered that the 2023 WHO Essential Medicines List (WHO EML) does not include any of these FDC products for the common cold; in fact, the only listed FDCs are those supported by strong pharmacokinetic and pharmacodynamic evidence, such as trimethoprim + sulphamethoxazole, certain beta-lactams + beta-lactamase inhibitors or antivirals.^16^

## Recommendations for action

The repeated DEG crises seem quite the Sisyphean work, and the inability to avoid new cases once the causes have been identified recalls a sort of understandable absurd. Given the situation, in our opinion, independently of the compulsory and strict inspections of manufacturers to detect any human error or negligence, the discussion should be centred on the early detection of any new case and, especially, reducing chances for new episodes to happen. To mitigate the harm produced by substandard products in general and those contaminated with DEG, the best response consists in:

- Strengthening pharmacovigilance systems in low- and middle-income countries (LMIC). Around 170/190 countries are already engaged in the WHO Programme for International Drug Monitoring, although it should be accepted that not all are well prepared or have the adequate means for a quick response.
- Empowering patients to report unexpected outcomes after receiving pharmacological treatment and promoting responsible self-medication, which means basic healthcare education and engaging them actively in the reporting system in place in their country.
- Perhaps the most important way to start active prevention, although it requires strong political commitment, good pharmacological knowledge and transparency, is to start revising the products holding marketing authorisation considering the present evidence supporting their benefit-risk balance of FDC products to avoid patients receiving useless medicines that, additionally could cause harm.
- Promoting an agreement among manufacturers to introduce a detection mechanism of DEG (a pungent odour or an inert permanent dye) as an initial screening method to avoid contamination errors during the manufacturing and handling process. Additionally, we advocate for the integration of more accurate and sensitive methods, such as Thin Layer Chromatography (TLC) or Gas Chromatography-Mass Spectrometry (GC-MS), to ensure reliable and definitive detection of DEG contamination, safeguarding the quality and safety of pharmaceutical products.
- Historically, quality assurance emphasised end-product testing; however, the intricate nature of the pharmaceutical supply chain highlights the necessity for increased regulatory emphasis on robust quality controls for testing and analysing raw materials, active pharmaceutical ingredients (APIs), and excipients.

## Conclusion

In conclusion, the ongoing incidents of DEG contamination – particularly those related to irrational fixed-dose combinations – underscore a crucial opportunity to enhance quality control measures and reassess the marketing authorisations of products lacking evidence-based support. Aligning national medicine lists and clinical guidelines with WHO recommendations can significantly strengthen these initiatives. Furthermore, there is an urgent need to reinforce regulatory systems, especially in African countries, focusing on both pre-production and post-marketing phases. This includes establishing robust pharmacovigilance systems to track adverse drug reactions after market introduction for early detection of incidence, as well as implementing regular monitoring of pharmaceutical products. Ultimately, a comprehensive reform of the global pharmaceutical market is vital to prioritise evidence-based medicine, enforce stringent quality control and ensure effective regulatory oversight.
